# Augmented Reality–Based Rehabilitation of Gait Impairments: Case Report

**DOI:** 10.2196/17804

**Published:** 2020-05-26

**Authors:** Jeremia Philipp Oskar Held, Kevin Yu, Connor Pyles, Janne Marieke Veerbeek, Felix Bork, Sandro-Michael Heining, Nassir Navab, Andreas Rüdiger Luft

**Affiliations:** 1 Division of Vascular Neurology and Neurorehabilitation Department of Neurology University of Zurich and University Hospital Zurich Zurich Switzerland; 2 University Hospital Klinikum rechts der Isar Technical University of Munich Munich Germany; 3 Johns Hopkins Applied Physics Laboratory Johns Hopkins University Laurel, MD United States; 4 Computer Aided Medical Procedures and Augmented Reality Technical University of Munich Munich Germany; 5 Department of Trauma Surgery University of Zurich and University Hospital Zurich Zurich Switzerland; 6 Computer Aided Medical Procedures Johns Hopkins University Baltimore, MD United States; 7 cereneo Center for Neurology and Rehabilitation Vitznau Switzerland

**Keywords:** HoloLens 2, gait, rehabilitation, stroke, augmented reality, sensors

## Abstract

**Background:**

Gait and balance impairments are common in neurological diseases, including stroke, and negatively affect patients’ quality of life. Improving balance and gait are among the main goals of rehabilitation. Rehabilitation is mainly performed in clinics, which lack context specificity; therefore, training in the patient’s home environment is preferable. In the last decade, developed rehabilitation technologies such as virtual reality and augmented reality (AR) have enabled gait and balance training outside clinics. Here, we propose a new method for gait rehabilitation in persons who have had a stroke in which mobile AR technology and a sensor-based motion capture system are combined to provide fine-grained feedback on gait performance in real time.

**Objective:**

The aims of this study were (1) to investigate manipulation of the gait pattern of persons who have had a stroke based on virtual augmentation during overground walking compared to walking without AR performance feedback and (2) to investigate the usability of the AR system.

**Methods:**

We developed the ARISE (Augmented Reality for gait Impairments after StrokE) system, in which we combined a development version of HoloLens 2 smart glasses (Microsoft Corporation) with a sensor-based motion capture system. One patient with chronic minor gait impairment poststroke completed clinical gait assessments and an AR parkour course with patient-centered performance gait feedback. The movement kinematics during gait as well as the usability and safety of the system were evaluated.

**Results:**

The patient changed his gait pattern during AR parkour compared to the pattern observed during the clinical gait assessments. He recognized the virtual objects and ranked the usability of the ARISE system as excellent. In addition, the patient stated that the system would complement his standard gait therapy. Except for the symptom of exhilaration, no adverse events occurred.

**Conclusions:**

This project provided the first evidence of gait adaptation during overground walking based on real-time feedback through visual and auditory augmentation. The system has potential to provide gait and balance rehabilitation outside the clinic. This initial investigation of AR rehabilitation may aid the development and investigation of new gait and balance therapies.

## Introduction

Many neurological diseases lead to impairments of gait and balance [1]. Approximately 80% of persons who have a stroke experience such deficits [2]. The key characteristics of impaired gait after stroke are shortened stance phase of the affected side, reduced knee and hip flexion during swing, slower walking speed, and shorter stride length [3]. Balance impairments are characterized by reduction in maximum weight shift toward the affected side [4], delayed postural reactions [5], shift of the center of mass toward the non-affected side, and decreased ability to avoid obstacles [6]. These gait and balance impairments are increased when patients are required to perform a cognitive task in parallel [7] and in patients who have attention or vision deficits [8,9]. Impaired gait and balance poststroke can have profound consequences for patients, as these impairments are strongly related to increased fear of falling [10] and reduced quality of life [11].

Therefore, improving gait and balance is one of the main goals of stroke rehabilitation. Repetitive practice is an essential ingredient of established evidence-based interventions [12] such as speed-dependent treadmill training, postural control with visual feedback training, and task-specific training [13]. These interventions can be delivered as part of inpatient or outpatient programs. In all these settings, training lacks context specificity (i.e., mobility in the daily life environment of the individual patient). In the last decade, virtual reality (VR) training systems have been found to effectively improve gait and balance after stroke [14,15]. VR systems provide challenging training situations and many different training environments. However, these VR systems still do not reflect the real-world environment of the patient; also, they require expensive stationary equipment. In addition, these systems are often limited in their variety of exercises to improve balance during gait training [16]. To interact with the real-world environment, augmented reality (AR) is an option to provide multiple sensory feedback enhanced by computer-generated perceptual information.

Considering the importance of performance feedback for motor skill learning [17], AR combined with sensor-based kinematic measurements can deliver fine-grained visual and auditory feedback on gait and balance parameters; this feedback can provide higher specificity and continuity and lower delay than the feedback delivered by a therapist [18]. Sensor-based motion capture systems, including inertial measurement units, can measure a patient’s gait kinematics and center of mass outside the laboratory and clinic [19], although laboratory-based optical tracking systems remain the gold standard with respect to the sensitivity and accuracy of these systems [20].

Here, we propose a new method for gait and balance rehabilitation in patients who have had a stroke that has potential to provide challenging and personalized gait and balance therapy with auditory and visual performance feedback based on gait kinematics in an environment that is adjusted to the patients’ needs. We developed the Augmented Reality for gait Impairments after StrokE (ARISE) system, in which we combined a development version of a head-mounted system for real-time visual and auditory feedback and a commercially available sensor-based motion capture system. Subsequently, we evaluated the usability of the AR feedback prototype in a chronic stroke subject with minor gait and balance impairments. We hypothesized that this system is more capable of modifying gait kinematics than walking without the system.

## Methods

### Ethics Statement

Ethical clearance to execute the experiment with the presented subject was provided by the Cantonal Ethics Commission of the Canton of Zurich, Switzerland (BASEC-Nr. Req-2019-00758). The participant received information about the experiments. Written informed consent in accord with the Declaration of Helsinki was obtained from the participant prior to performing the experiments.

### Patient Information

The patient was a right-handed man aged 74 years who had a right-hemispheric ischemic stroke in the thalamus, capsula interna, and right temporal lobe 7 years before participating in the experiment. Acutely after stroke, he had mild motor deficits in the left leg (National Institutes of Health Stroke Scale leg item score 1/4). At the time of this experiment, he had minor limitations of sitting and standing balance (Berg Balance Scale 54/56); he also had mild limitations in motor function (Fugl-Meyer Motor Assessment lower extremity subscale 29/34) and strength (Motricity Index lower extremity subscale 88/100) of the affected left side. The patient walked independently without a walking aid (Functional Ambulation Categories 5/5), with a comfortable walking speed of 1.0 m/s and a step length of 0.56 meters as measured by the 10-Meter Walk Test. The patient had slight risk of falling (Dynamic Gait Index 19/24). He reported numbness in the feet and a feeling of wearing socks when he was not. In addition, a mild cognitive impairment was present (Montreal Cognitive Assessment 25/30).

### Materials

**Figure 1:**
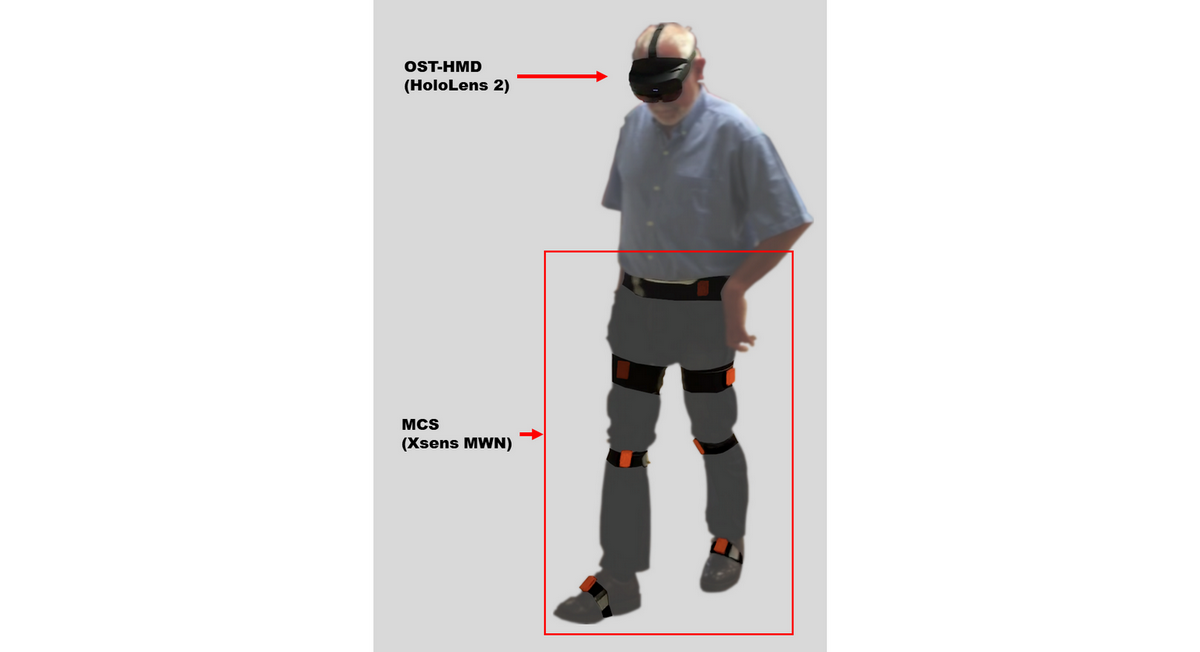
Patient who has had a stroke wearing the ARISE system, including the optical see-through head-mounted display (HoloLens 2) and the sensor-based motion capture system (Xsens MVN).

The ARISE system consisted of two essential components: an optical see-through head-mounted display (OST-HMD) and a sensor-based motion capture system (Figure 1, Supplementary Material A).

**Figure 2:**
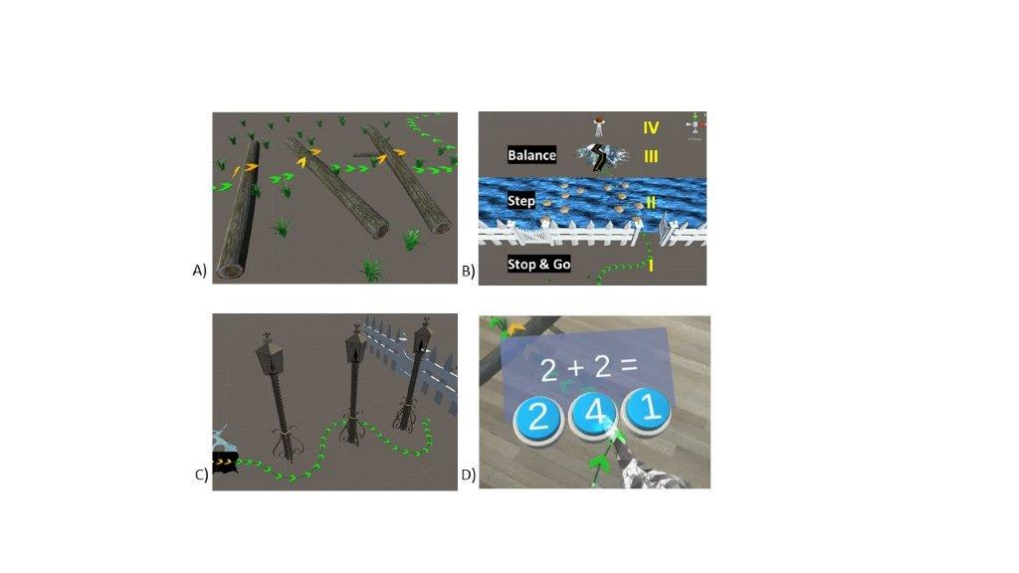
Augmented reality parkour course, including arrows indicating the walking direction. A) Overstep obstacle of tree trunks. B) I. Stop and go barrier; II. stepping stones over a virtual river; III. walkable ridge-path; IV. the patient turns around and walks back. C) Walking slalom with lamps. D) Dual-task math calculation.

We identified the HoloLens 2 (Microsoft Corporation) as the most suitable OST-HMD, as it provides a wider field of view (43×29 degrees) compared to other devices and thus can display more virtual objects in a real-world environment. In addition, the HoloLens 2 is able to track head movements with an inertial measurement unit and has an intuitive hand-interaction user interface that is enabled through fully articulated hand tracking. The OST-HMD was used to visualize the AR parkour course (Figure 2 A-C). The state-of-the-art parkour course had an area of approximately 14×4 meters; it consisted of visualizations of real-life obstacles and barriers, such as blocks and floor mats, that forced the patient to perform certain leg movements. A trail of arrows indicated the walking direction, and the parkour course changed dynamically depending on the position of the patient.

In addition, the OST-HMD provided visual and auditive feedback based on real-time gait kinematic performance. An inertial measurement unit–based motion capture system, the Xsens MVN (Xsens Technologies B.V.), was chosen to track the kinematics of the lower limbs during gait. We strapped seven inertial measurement units on the pelvis and the lower extremity of the patient (Figure 1). The Xsens software (MVN version 2019.2) converted the rotational data from the inertial measurement units into a fully articulated virtual mannequin. This provided translational and rotational data of every large humanoid joint. With this combination, the patient was able to walk longer distances (more than 10 meters).

The HoloLens 2 and Xsens systems were integrated by streaming movement performance data to the OST-HMD through a user datagram protocol client. To adjust for translational drift of the motion capture system in the ARISE system, the captured motion data were rigidly attached to the pose of the HoloLens 2. To synchronize the forward directions of both systems, we performed a short calibration phase where the user was required to align his head with his hips and feet. Once the 2 systems were coupled, we were able to provide subtle positive reinforcement of successful knee flexion (>45 degrees) by playing an ambient bird song sound through the HMD.

To increase the difficulty of the tasks the subject was asked to complete, simple math calculations were presented visually (dual task procedure, Figure 2 D). The subject responded by pressing a virtual button, which was detected by the hand-tracking capabilities of the HoloLens 2. At the end of the parkour course, a knowledge of results display was shown, including the time to run the parkour course, the correct answers to the math calculations, and the percentage of time that the condition of knee flexion >45 degrees was fulfilled.

We provided the participant with information about the experimental setup. After clinical assessments of the participant were performed, the sensors were fitted to his legs and pelvis, and he donned the HoloLens 2. First, a patient-personalized height scaling model was applied to the motion capture software. The motion capture system was calibrated. The participant performed a 10-meter walk test and then completed the AR parkour course three times. During walking, the 10-meter walk test and the AR parkour, the patient’s lower extremity kinematics and center of mass were tracked with the motion capture system. After the AR parkour, the System Usability Scale [21], the Virtual Reality Symptom Questionnaire [22], and a semi-structured interview were used to assess the patient’s experience using the AR system.

### Data Availability

All datasets generated for this publication are included in the manuscript.

## Results

**Figure 3:**
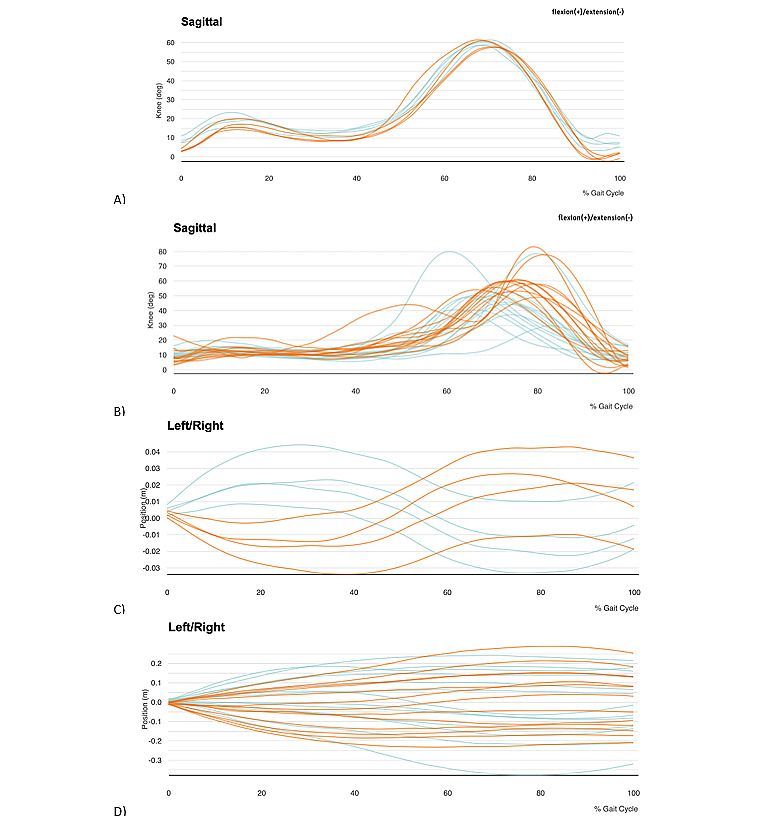
Knee flexion angles during gait cycles during A) a 10-meter walking test and B) the AR parkour. Orange lines represent the right knee, and blue lines represent the left knee. Center of mass position during gait cycles during C) the 10-meter walking test and D) the AR parkour. Orange lines represent the right leg, and blue lines represent the left leg.

The kinematic parameters of the knees and the center of mass while walking during the 10-meter walk test and the AR parkour are listed in detail in Table 1 and Table 2, respectively. The patient adapted his gait performance and increased his knee flexion angles during the gait cycle when performing the AR parkour compared to when walking a straight line during the 10-meter walk test (Figure 3). His walking speed in the 10-meter walk test did not change (pre 9 seconds, post 9 seconds).

During the AR parkour, the patient perceived the virtual objects, stepped over the obstacles and barriers, and reported a feeling of being in a real-world parkour course. Despite the positive effects on knee flexion, he did not consciously perceive the bird songs.

The patient overlooked the math problems during the first run but solved them in subsequent trials after being reminded to do so. This increased the time to complete the AR parkour from 75 seconds without the second task to 100 seconds with the second task.

The patient reported that wearing the HoloLens 2 felt like wearing a hat. He criticized the limited vertical field of view, which forced him to lean forward to see the obstacles directly beneath him (see the video in Multimedia Appendix 1). However, he ranked the usability of the AR parkour system as excellent (System Usability Scale 87.5/100). He stated that he would like to use the system more often for self-training to complement his current conventional outpatient therapy program. No adverse events were measured with the Virtual Reality Symptom Questionnaire, with the exception of the symptom of exhilaration.

**Table 1:** Kinematic parameters (joint angle, degrees) during the 10-meter walk test and the AR parkour.

Parameter			Stance^a^		Swing^b^		At foot strike^c^	At foot release^d^
			Minimum	Maximum	Minimum	Maximum	Mean (SD)	Mean (SD)
**Ten-meter walk test**								
	**Hip angle flexion**							
		Left^e^	–7.17	31.06	–3.20	33.57	28.34 (2.02)	–0.78 (0.54)
		Right	–11.18	29.63	–8.01	30.21	26.74 (0.84)	–4.00 (2.79)
		Difference	4.01	1.44	4.81	3.36	1.60	3.23
	**Knee angle flexion**							
		Left^e^	7.18	37.82	3.05	61.37	8.44 (1.59)	41.84 (0.38)
		Right	2.53	41.45	–2.50	61.34	3.04 (0.73)	38.23 (4.77)
		Difference	4.65	–3.63	5.55	0.03	5.40	3.61
	**Ankle angle flexion**							
		Left^e^	–4.92	19.49	–10.85	9.07	0.86 (1.19)	–4.05 (2.50)
		Right	–8.11	21.58	–26.09	7.63	–3.14 (2.25)	–4.62 (4.02)
		Difference	3.20	–2.09	15.24	1.44	4.00	0.57
**Augmented reality parkour**								
	**Hip angle flexion**							
		Left^e^	–2.09	29.77	–2.06	49.26	23.77 (3.42)	3.82 (4.80)
		Right	–3.55	33.90	–3.30	50.98	23.49 (4.68)	1.83 (5.40)
		Difference	1.45	–4.12	1.25	–1.72	0.27	1.98
	**Knee angle flexion**							
		Left^e^	5.26	28.28	5.63	79.74	10.22 (2.84)	23.06 (4.39)
		Right	3.00	43.83	–2.57	82.93	8.57 (5.42)	28.08 (6.04)
		Difference	2.26	–15.55	8.19	–3.19	1.65	–5.02
	**Ankle angle flexion**							
		Left^e^	–6.70	23.22	–4.49	21.33	3.60 (3.67)	15.28 (4.60)
		Right	–6.32	31.45	–16.95	29.63	2.13 (3.41)	17.48 (6.35)
		Difference	–0.38	–8.24	12.46	–8.31	1.47	–2.20

^a^Period of time from a foot strike to the following release of the same foot.

^b^Period of time from a foot release to the following strike of the same foot.

^c^Time at which the foot contacts the ground after a swing phase.

^d^Time at which the foot stops being in contact with the ground after a stance phase.

^e^The patient’s affected side.

**Table 2:** Position of the center of mass in meters during the 10-meter walk test and the augmented reality parkour.

Position			Stance^a^				Swing^b^				Overall			
			Minimum		Maximum		Minimum		Maximum			Minimum		Maximum
**Ten-meter walk test**														
	Left^c^			–0.02		0.04		–0.03		0.02			–0.03	0.04
	Right			–0.03		0.02		–0.02		0.04			–0.03	0.04
	Difference			0.01		0.02		–0.01		–0.02			0.00	0.00
**Augmented reality parkour**														
		Left^c^	–0.30		0.24		–0.38		0.24			–0.38		0.24
		Right	–0.23		0.26		–0.23		0.29			–0.23		0.29
		Difference	–0.07		–0.02		–0.15		0.05			–0.15		–0.05

^a^Period of time from a foot strike to the following release of the same foot.

^b^Period of time from a foot release to the following strike of the same foot.

^c^The patient’s affected side.

## Discussion

### Principal Findings

Gait and balance training requires training systems that are mobile and preferably wearable. The ARISE system proposed here combines flexible training with performance feedback in a wearable form by integrating an OST-HMD and a sensor-based motion capture system. It enables context-specific training and can display virtual objects via the HMD in a real-world space. The AR rehabilitation system can be used for overground walking, while gait kinematics are measured with the motion capture system. The combination of these technologies is new in gait rehabilitation. Most AR and VR systems consist of stationary equipment, including a treadmill, cameras, and projection devices [6]. Hence, they offer fewer options for adaptation [23] and are restricted to the unreal training environment of treadmill walking; moreover, a minimum of 6 minutes (around 400 strides) is required to achieve stable performance on a treadmill [24].

The ARISE system was tested during a single session experiment with one patient who had a stroke. The patient perceived the system to be comfortable, and it did not restrict his movements. The limited vertical field of view (29 degrees) of the HoloLens 2 should be improved to increase the usability of the system. The field of view forced the patient to bend forward; this can induce adverse events, such as near-falls or neck pain, when using the system over longer periods of time.

Through the coupling of the OST-HMD and the motion capture system, the patient perceived auditive feedback based on the kinematics of the knee (flexion angle). The OST-HMD provided feedback in the form of bird songs and a knowledge of results display. When the patient was given multimodal feedback, he changed his gait pattern. This is in line with previous results [6,20]. A transfer of training from the ARISE system to overground walking can be expected when increasing the duration of the intervention [13]. Consolidation may be further improved by adding monetary rewards [25].

The current AR parkour course has a size of 14×4 meters; therefore, it requires a large open space to operate. In future versions, the ARISE system will be able to generate a parkour course with an adaptable area depending on the local environment. This will provide the advantages of flexibility in choosing the test grounds and a variety of parkour designs. In future research, the impacts of different virtual obstacles or even obstacle themes (e.g., street, garden, supermarket) on the gait kinematics can be evaluated. Due to the high mobility of all involved components, our system can be used virtually anywhere, both indoors and outdoors and with different surfaces and distractors. The mobility of the system is also ensured through the web interface of the OST-HMD and the motion capture system, which allows remote access. Therefore, a telerehabilitation approach is conceivable.

The present ARISE system is a prototype, and it requires technical support for setup and calibration. Therefore, a reduced sensor set and simplified calibration procedure would increase its usability and applicability. Furthermore, movement sensors are limited by orientation drift when they are used over a long period of time [26]. In this study, we did not use the system long enough to observe this drift. These issues of technical support and drift currently limit the use of the system in telerehabilitation and should be addressed in future studies [27].

### Conclusions

The ARISE system combines an adjustable and personalized training environment with a feedback and monitoring system for gait and balance rehabilitation. This case study showed that use of the system by patients who have had a stroke and who have mild gait and balance impairments is promising. Compared to existing AR systems, the system utilizes the real physical environment to place virtual obstacles that are immediately and intuitively treated as real-world objects. This system can be used to provide feedback on a variety of gait parameters and to implement personalized (dual-task) gait training environments.

## Appendix

Multimedia Appendix 1 Augmented Reality parkour for gait Impairments after StrokE.

## Abbreviations

AR: augmented reality
ARISE: Augmented Reality for gait Impairments after StrokE
OST-HMD: optical see-through head-mounted display
VR: virtual reality
